# cGAS-STING Pathway-Induced BST2 Enhances HPV-Infected Keratinocyte Proliferation in Condyloma Acuminata

**DOI:** 10.3390/biomedicines14020339

**Published:** 2026-02-01

**Authors:** Huayu Huang, Lian Liu, Xiaohang Xie, Yuchun Cao, Zhichao Gu

**Affiliations:** Department of Dermatology, Tongji Hospital, Tongji Medical College, Huazhong University of Science and Technology, Wuhan 430030, China

**Keywords:** condyloma acuminata, keratinocytes, cell proliferation, bone marrow stromal cell antigen 2 (BST2)

## Abstract

**Background**: Condyloma acuminata (CA) is a common sexually transmitted disease caused by human papillomavirus (HPV). Abnormal keratinocyte proliferation is a hallmark of CA, but the underlying mechanisms remain unclear. BST2, an interferon-stimulated gene, is implicated in viral inhibition and tumor cell proliferation. This study aimed to investigate whether BST2 is involved in HPV-induced keratinocyte proliferation. **Methods**: We conducted bioinformatics analysis using publicly available datasets from the Gene Expression Omnibus (GEO) to assess BST2 expression in CA. HPV-6/11 live virus and HPV11-E7 lentiviruses were used to infect HaCaT cells to mimic early HPV infection and viral genome integration. We examined BST2 expression in both CA patient tissue samples and in vitro models using RT-qPCR, Western blot, and immunohistochemistry. To investigate the signaling mechanisms, we used siRNA to knock down key components of the cGAS/STING pathway and examined BST2 expression levels. Additionally, we assessed keratinocyte proliferation through CCK-8 assays and cell counting. Activation of downstream signaling pathways was evaluated using Western blot analysis for key molecules in the MEK/ERK/c-Myc pathway. **Results**: BST2 was significantly upregulated in CA lesions and HPV-infected keratinocytes through the cGAS/STING pathway. BST2 activation promoted keratinocyte proliferation via the MEK/ERK/c-Myc pathway, and this effect was significantly inhibited by BST2 knockdown. **Conclusions**: HPV could promote the proliferation of keratinocytes from condyloma acuminata lesions through inducing BST2, indicating that BST2 would be a potential therapeutic target for condyloma acuminata.

## 1. Introduction

Low-risk human papillomavirus (HPV) types, such as HPV-6 and HPV-11, are associated with benign lesions like condyloma acuminata (CA) and do not typically cause cancer. In contrast, high-risk HPV types are linked to the development of malignant lesions such as cervical cancer. Condyloma acuminata (CA) is a common sexually transmitted disease primarily caused by low-risk HPV. In addition to sexual transmission, HPV can be transmitted through several other routes, including vertical transmission during childbirth, autoinoculation (self-infection), non-sexual skin-to-skin contact, and through contact with objects contaminated with the virus (fomites) [1]. It is characterized by abnormal proliferation of keratinocytes, leading to the formation of typical warts on the anogenital skin and mucosal tissues. Despite extensive research, no specific drugs effectively target HPV infection and replication [2]. Consequently, current clinical management of CA focuses on wart removal, with common treatments including cryotherapy, high-frequency electrosurgery, CO_2_ laser, and photodynamic therapy [3,4]. However, these approaches primarily address symptoms and fail to target the underlying molecular mechanisms involved in wart development. Consequently, residual HPV in the treated warts and surrounding sub-clinically infected areas often lead to high recurrence rates [5,6]. Thus, it is essential to further explore the pathogenesis of CA and develop novel therapeutic targets.

Innate immunity serves as the body’s initial defense against external viral infections, with IFN-mediated antiviral effects playing a key role [7,8]. The capsid protein L1 and L2 of low-risk HPV facilitate viral entry by binding to host cell membrane receptors, leading to endocytosis of viral particles. Once internalized, viral particles enter multivesicular bodies, where the capsid proteins are cleaved, exposing the vDNA, a pathogen-associated molecular pattern (PAMP). This vDNA is recognized by pattern recognition receptors (PRRs) within the host cell, initiating the Cyclic GMP–AMP Synthase (cGAS)/Stimulator of Interferon Genes (STING) signal pathway and triggering the production of IFNs [8,9,10]. These IFNs bind to receptors on the surface of target cells, activating the downstream JAK/STAT signal pathway. This activation drives the transcription and translation of numerous interferon-stimulated genes (ISGs) within the target cells, ultimately exerting an antiviral effect [11,12].

The MEK/ERK signaling cascade is a key regulator of cellular processes such as proliferation, survival, and differentiation. Upon activation, mitogen-activated protein kinase kinase (MEK) phosphorylates extracellular signal-regulated kinase (ERK), which then translocates to the nucleus and activates transcription factors such as c-Myc. c-Myc is a crucial regulator of cell cycle progression and metabolism, and its overexpression is often associated with tumorigenesis [13,14]. In the context of HPV infection, dysregulation of the MEK/ERK/c-Myc pathway contributes to abnormal keratinocyte proliferation, a hallmark of CA.

Tetherin, also called bone marrow stromal cell antigen 2 (BST2), is a type II transmembrane protein. BST2 was initially identified for its high expression on the surface of malignant plasma cells in the bone marrow and peripheral blood of multiple myeloma patients, while being expressed at lower levels in other normal tissues [15,16]. Subsequent research has revealed that increased BST2 is also closely associated with the excessive proliferation of tumor cells in various solid tumors such as breast cancer, pancreatic cancer, gastric cancer, and others [17,18,19].

In this study, we found that BST2 is significantly increased in keratinocytes from CA lesions. Further study has found that low-risk HPV can effectively induce the expression of BST2 through cGAS/STING signaling pathway in keratinocytes. Then we further demonstrated that increased BST2 can effectively promote the proliferation of keratinocyte from CA lesions. Mechanistically, enhanced BST2 can effectively activate the MEK/ERK/c-Myc signal pathway, thereby boosting the proliferation of keratinocytes.

## 2. Materials and Methods

### 2.1. Patients and Normal Donor Specimens

The diagnosis of condyloma acuminata (CA) was based on typical clinical manifestations and HPV genotyping tests conducted at the Department of Dermatology, Tongji Hospital (Wuhan, China). A total of 89 condyloma acuminata specimens were collected, along with 47 healthy control foreskin specimens. These specimens were obtained from the Pathology Research Unit of the Department of Dermatology, Tongji Hospital, with informed consent obtained from all patients and normal donors. This was a prospective study. The study was approved by the Institutional Review Board of Tongji Hospital (approval number: TJ-IRB20211206) on 2 December 2021.

### 2.2. Cell Culture and Tissue Samples

The human immortal keratinocyte HaCaT cells were purchased from the China Center for Type Culture Collection (CCTCC, Wuhan, China). The human embryonic kidney 293FT cells were purchased from the Cell Resource Center, Peking Union Medical College (PCRC, Beijing, China). HaCaT cells were cultured in DMEM (0.03 mM Ca^2+^) supplemented with 10% FBS, 1% penicillin/streptomycin in a 5% CO_2_ incubator at 37 °C. 293FT cells were cultured in DMEM supplemented with 10% FBS, 1% penicillin/streptomycin and 1% NEAA in a 5% CO_2_ incubator at 37 °C. Before the trial began, HaCaT cells and 293FT cells were checked for mycoplasma, interspecies cross-contamination, and authenticity using short tandem repeat profiling and isoenzyme analysis. The culture of HaCaT cells was kept for a maximum of 15 passages.

### 2.3. RNA Interference

The siRNAs targeting the targeted genes and a negative control siRNA were purchased from RiboBio (Guangzhou, China). Details of the gene sequences are shown in Table S1. Cells were transfected with the siRNAs using Lipofectamine 3000 (Invitrogen, Carlsbad, CA, USA). The silencing efficiency was verified by real-time PCR and Western blot.

### 2.4. Cell Counting

HaCaT cells were divided into groups and inoculated in six-well plates with two replicate wells. Cell counts were taken at 24 h, 48 h, and 72 h using a hematocyte counter. Each well was counted three times and the average value was used for analysis.

### 2.5. Cell Counting Kit 8 (CCK-8) Assay

Cell Counting Kit-8 (CCK-8; MedChemExpress, Monmouth Junction, NJ, USA) was utilized to perform the CCK-8 assay. HaCaT cells were seeded into 96-well plates with 4 × 10^3^ cells/well at 37 °C. After incubation for indicated time points, 10 μL of CCK-8 reagents were added into the wells. The whole process needs to avoid light. 96-well plates were incubated at 37 °C and 5% CO_2_ for 1 h. Finally, the optical density values were detected at 450 nm by enzyme-labeled instrument.

### 2.6. Cell Apoptosis Detection

The apoptoses of different HaCaT cell groups were detected with an Annexin-V/PI Apoptosis Detection Kit (KeyGen Biotech, Nanjing, China) according to the manufacturer’s protocol and then analyzed using an NovoCyte Flow Cytometer Systems 1–3 Lasers (Agilent Technologies, Santa Clara, CA, USA).

### 2.7. Real-Time PCR

Total RNA of sample cells was extracted using the TRIzol reagent (Invitrogen, Carlsbad, CA, USA) following the manufacturer’s protocols. Real-time PCR analysis was performed using the SYBR Green PCR mix (TOYOBO, Osaka, Japan) on a CFX Connect Real-Time PCR Detection System (Bio-Rad, Hercules, CA, USA). Comparative quantitative mRNA levels of cells were normalized to the housekeeping gene β-actin. Primers for real-time PCR were synthesized in Tsingke Biotechnology (Beijing, China). The primer sequences were shown in Table S2.

### 2.8. Immunohistochemistry

We prepared wax blocks of tissue specimens according to the above operation and performed serial sectioning. Before staining, the sections were subjected to antigen retrieval in sodium citrate buffer (0.01 M, pH 6.0) at 100 °C for 15 min and incubated with 10% normal goat serum for 30 min, followed by incubation overnight at 4 °C with commercial rabbit monoclonal antibodies against BST2 (Cell Signaling Technology, Danvers, MA, USA, # 95940T) at 1/200 dilution. Then, the sections were conjugated with Goat Anti-Rabbit IgG H&L (1:500 dilution; Servicebio, Wuhan, China) at room temperature for 2 h, then covered by diaminobenzidine (DAB) (Servicebio, Wuhan, China). Subsequently, all fields were observed under light microscopy (NanoZoomer S360, Hamamatsu Photonics, Hamamatsu, Japan). Control experiments without primary antibody demonstrated that the signals observed were specific.

### 2.9. Western Blot Analysis

Cell samples were lysed using a combination of RIPA, a protease inhibitor, and a phosphatase inhibitor. Antibodies for Western blot analysis included: anti-GAPDH (Abbkine, Wuhan, China), anti-β-Tubulin (Abbkine, Wuhan, China), anti-BST2 (Cell Signaling Technology, Danvers, MA, USA), anti-STING (Cell Signaling Technology, Danvers, MA, USA), anti-STING (phospho S366) (Cell Signaling Technology, Danvers, MA, USA), anti-IRF-3 (Cell Signaling Technology, Danvers, MA, USA), anti-IRF-3 (phosphor S386) (Cell Signaling Technology, Danvers, MA, USA), anti-MEK1 + MEK2 (Abcam, Cambridge, UK), anti-MEK1 + MEK2 (phospho S218 + S222) (Abcam, Cambridge, UK), anti-ERK1 + ERK2 (Abcam, Cambridge, UK), anti-ERK1 (phospho T202) + ERK2 (phospho T185) (Abcam, Cambridge, UK), and anti-c-Myc (Proteintech, Rosemont, IL, USA) at the recommended dilutions.

### 2.10. Low-Risk HPV Extraction and Infection

Freshly isolated genital wart tissues obtained from clinical patients were first dissected and ground in a phosphate-buffered saline (PBS) solution. The tissue suspension was then subjected to centrifugation, and the resulting supernatant was collected. The obtained wart supernatant was confirmed to be HPV-6 or HPV-11 positive using a specific detection kit (DaAn Gene, Guangzhou, China) and was verified to be free of other high-risk HPV types. The supernatant was then used for subsequent experiments and incubated with HaCaT cells.

### 2.11. Statistical Analysis

Datasets from the Gene Expression Omnibus database (https://www.ncbi.nlm.nih.gov/geo/) (accessed on 20 December 2023) were analyzed using R software (v.4.4.0). All experimental data were analyzed by *t*-tests using GraphPad Prism 9.0 software, and then expressed as means ± SEM. Differences were regarded as statistically significant at *p* < 0.05.

## 3. Results

### 3.1. Bioinformatic Analysis of CA-Related Datasets from the GEO Database

**Figure 1:**
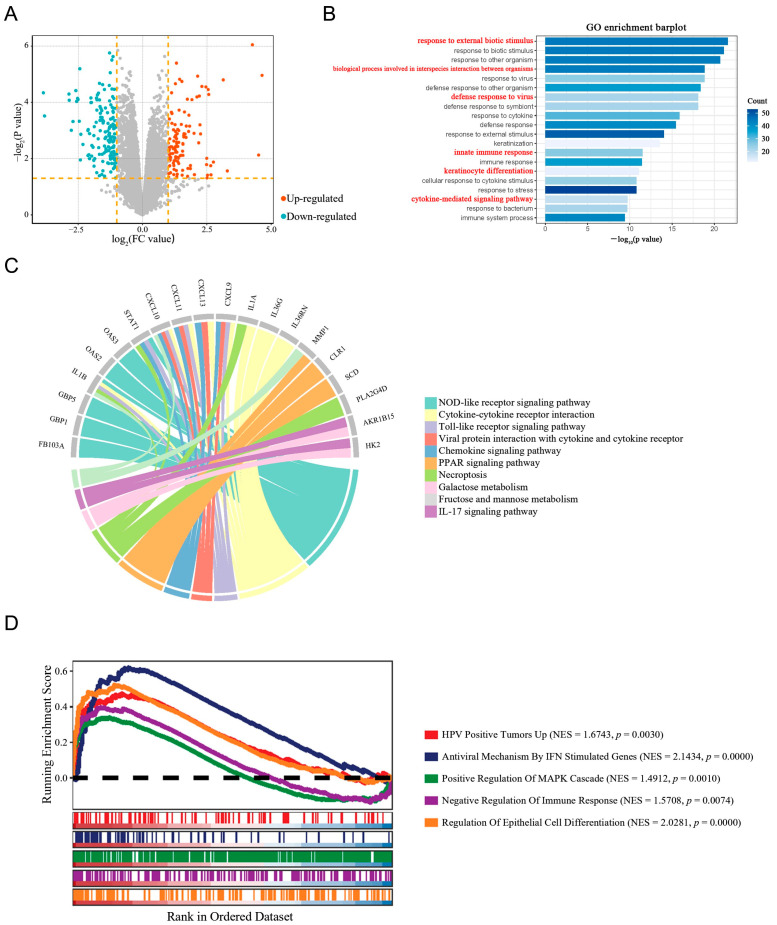
Differential expression analysis and functional enrichment of DEGs in CA. (**A**) Volcano plot of DEGs between CA and normal skin tissues from the GSE140662 dataset. Orange dots represent up-regulated DEGs, and blue dots represent down-regulated DEGs. Gray dots represent genes with no significant differential expression. (**B**) GO enrichment analysis of DEGs, highlighting immune-related biological processes. Bar length represents gene count, and color intensity reflects significance. The GO terms highlighted in red font indicate key interferon-stimulated genes (ISGs) of interest. (**C**) Chord diagram linking DEGs to key signaling pathways, with distinct colors representing different pathways. (**D**) GSEA results of selected gene BST2, with enrichment scores plotted against ranked gene sets. The dashed horizontal line marks Running Enrichment Score (ES) = 0, serving as the baseline to visualize positive vs. negative deviation of the running ES across the ranked gene list.

Previous studies have found that high-risk HPV infections, such as HPV-16, can suppress the expression of ISGs [19]. However, it remains unclear whether low-risk HPV infections result in the same outcomes as high-risk HPV infections. In our prior work, we showed that WTAP-mediated m6A methylation enhances IFN-ε expression in condyloma acuminata (CA) keratinocytes, strengthening local antiviral immunity [8]. However, the role of downstream ISGs remains unclear. To explore ISGs expression in CA warts, we analyzed our group’s prior transcriptome sequencing data (GSE140662) using the limma package in R to identify differentially expressed genes (DEGs). This analysis revealed 282 DEGs including 120 up-regulated DEGs and 162 down-regulated DEGs, which was partially shown in volcano plot (Figure 1A). Subsequently, Gene Ontology (GO) and Kyoto Encyclopedia of Genes and Genomes (KEGG) pathway enrichment analyses were performed to elucidate the functions of upregulated DEGs, with results displayed in bar plots and chord diagrams (Figure 1B,C). In this study, we focused on BST2 to investigate its potential involvement in CA development and progression. We performed GSEA analysis on BST2 to identify specific biological processes and associated signaling pathway (Figure 1D).

### 3.2. BST2 Is Induced in Keratinocytes from CA Warts

**Figure 2:**
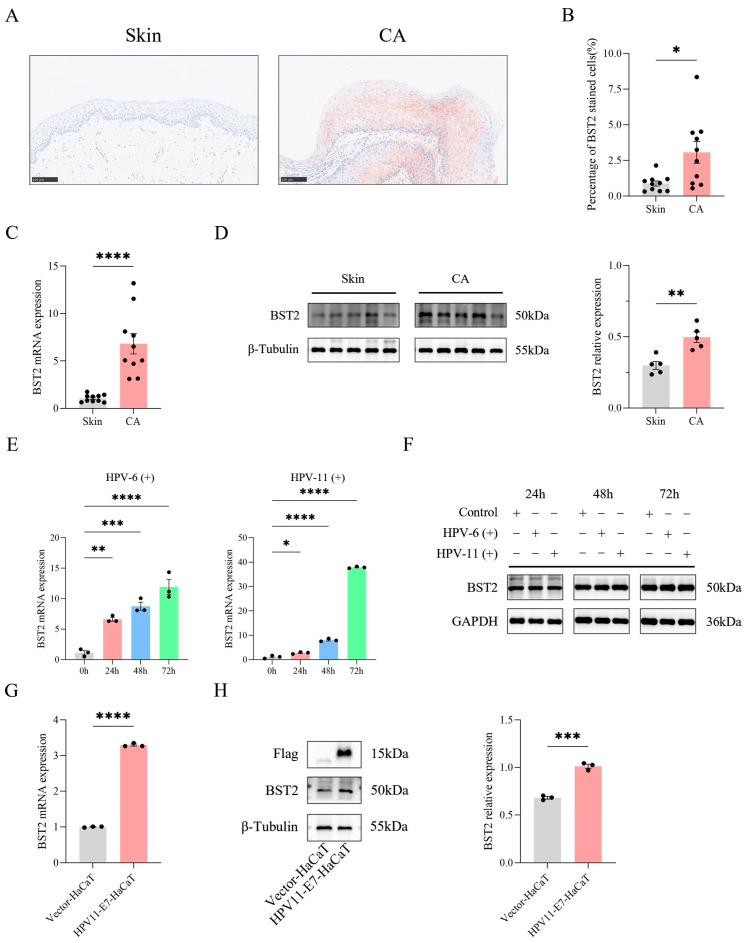
BST2 expression is upregulated in condyloma acuminatum (CA) and HPV-infected cells. (**A**) Immunohistochemical staining of BST2 in normal skin and CA tissues (Magnification: 200×). (**B**) Quantification of BST2-positive stained cells in skin and CA tissues, each dot represents one of 10 different clinical specimens. (**C**) qRT-PCR analysis of BST2 mRNA expression in skin and CA tissues, each dot represents one of 10 different clinical specimens. (**D**) Western blot analysis of BST2 protein expression in skin and CA tissues, with β-Tubulin as a loading control, each dot represents one of 5 different clinical specimens. (**E**) Time-course qRT-PCR analysis of BST2 mRNA expression in HPV-6(+) and HPV-11(+) infected HaCaT cells at 0 h, 24 h, 48 h, and 72 h. Each dot represents one of 3 independent replicate experiments. (**F**) Western blot analysis of BST2 protein levels in HPV-6(+) and HPV-11(+) infected HaCaT cells at different time points. GAPDH serves as a loading control. (**G**) qRT-PCR analysis of BST2 mRNA expression in cells transfected with vector-lentivirus or HPV11-E7-lentivirus. Each dot represents one of 3 independent replicate experiments. (**H**) Western blot analysis of BST2 expression in vector-HaCaT and HPV11-E7-HaCaT cells, with β-Tubulin as a loading control. The right panel shows quantification of BST2 protein levels, where each dot represents one of 3 independent replicate experiments. (* *p* < 0.05, ** *p* < 0.01, *** *p* < 0.001, **** *p* < 0.0001).

To validate bioinformatics findings, we infected HaCaT cells with wart-derived homogenate containing live low-risk HPV or lentiviruses expressing HPV11-E7 to investigate the early cell entry of HPV and biological events following vDNA integration into the host genome (Figure S1A,B). RT-qPCR analysis revealed significantly upregulated BST2 expression in both cell models compared to controls (Figure 2E,G), with consistent increases in BST2 protein levels (Figure 2F,H). Additionally, immunohistochemical staining of CA tissues and normal foreskin tissues showed minimal BST2 staining in keratinocytes of normal foreskin across the cell membrane, cytoplasm, and nucleus. In contrast, CA tissues displayed pronounced BST2 staining in keratinocyte cell membranes, with a significantly higher proportion of BST2-positive cells in the CA group compared to the normal skin group (Figure 2A,B). RT-qPCR and Western blot analyses of these tissues confirmed elevated BST2 expression in CA tissues (Figure 2C,D). Collectively, these findings demonstrate a marked increase in BST2 expression in CA-warts and HPV-infected cell models.

### 3.3. HPV Infection Induces BST2 Expression in Keratinocytes Through the cGAS/STING Signal Pathway

**Figure 3:**
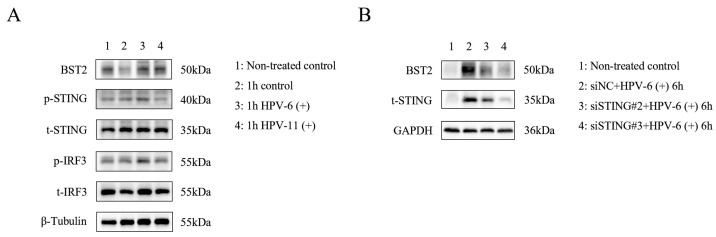
BST2 upregulation is associated with the STING pathway in HPV-infected cells. (**A**) Western blot analysis of BST2, phosphorylated STING (p-STING), total STING (t-STING), phosphorylated IRF3 (p-IRF3), and total IRF3 (t-IRF3) in control and HPV-6(+) or HPV-11(+) treated cells for 1 h. β-Tubulin was used as a loading control. (**B**) Western blot analysis of BST2 and total STING in cells transfected with siNC or siSTING and treated with HPV-6(+) for 6 h. GAPDH was used as a loading control.

Following stimulation of HaCaT cells with wart-derived homogenate containing live low-risk HPV virus for 1 h, Western blot analysis revealed a significant upregulation of BST2 protein expression compared to the control group, accompanied by increased phosphorylation levels of STING and IRF3 proteins (Figure 3A). To explore the mechanism, we silenced STING expression in HaCaT cells using STING-specific siRNA. Following HPV stimulation under the same conditions, BST2 expression was not significantly elevated in STING-deficient cells (Figure 3B). These findings suggest that low-risk HPV infection promotes BST2 expression in keratinocytes, likely mediated through activation of the cGAS/STING signaling pathway.

### 3.4. BST2 Promoted the Proliferation of Keratinocytes

**Figure 4:**
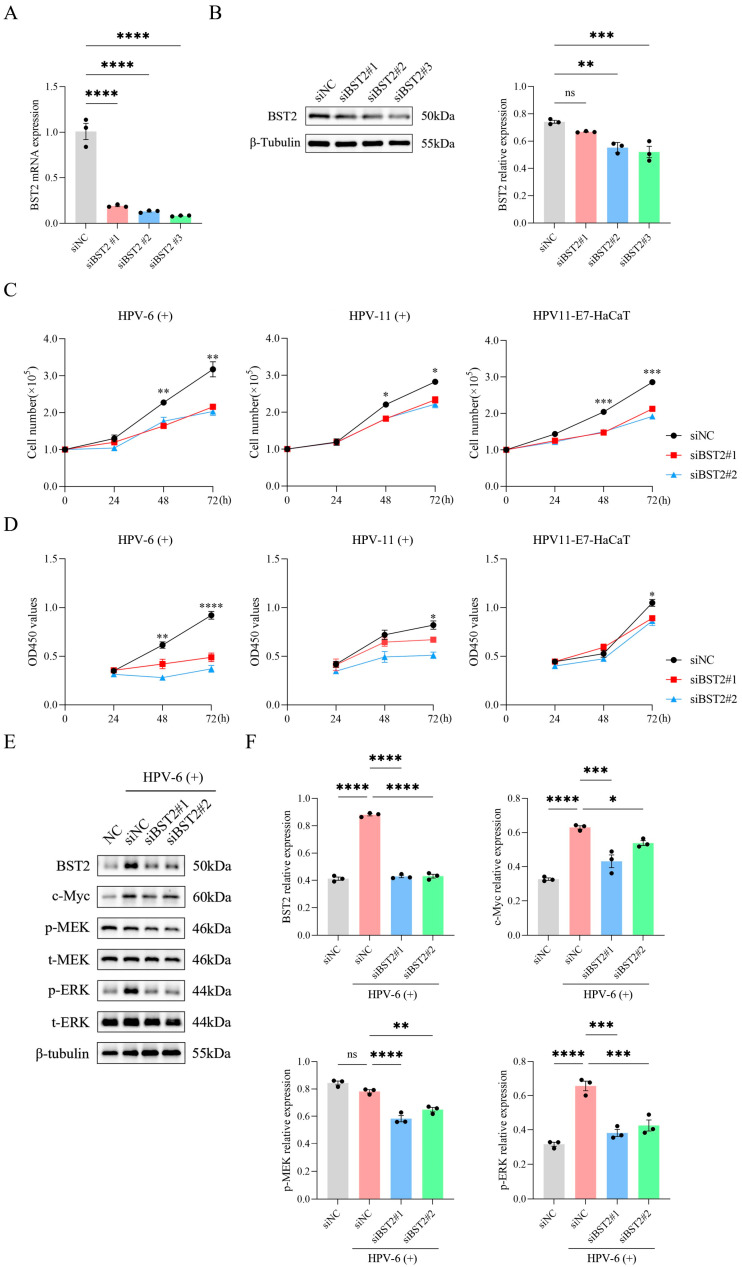
Knockdown of BST2 inhibits HPV-induced cell proliferation via the MEK/ERK signaling pathway. (**A**) qRT-PCR analysis of BST2 mRNA expression in cells transfected with siNC or three different BST2 siRNAs (siBST2#1, siBST2#2, siBST2#3), each dot represents one of 3 independent experiments. (**B**) Western blot analysis and quantification of BST2 protein expression after BST2 knockdown. β-Tubulin was used as a loading control, each dot represents one of 3 independent experiments. (**C**) Cell proliferation assays in HPV-6(+), HPV-11(+), and HPV11-E7-HaCaT cells transfected with siNC or siBST2 (siBST2#1, siBST2#2) for 24, 48, and 72 h. (**D**) CCK-8 assays measuring cell viability at 24, 48, and 72 h post-transfection in HPV-6(+), HPV-11(+), and HPV11-E7-HaCaT cells. (**E**) Western blot analysis of BST2, c-Myc, phosphorylated MEK (p-MEK), total MEK (t-MEK), phosphorylated ERK (p-ERK), and total ERK (t-ERK) in HPV-6(+) cells transfected with siNC or siBST2 (siBST2#1, siBST2#2). β-Tubulin was used as a loading control. (**F**) Quantification of BST2, c-Myc, p-MEK, and p-ERK protein expression levels from (**E**), each dot represents one of 3 independent experiments. (* *p* < 0.05, ** *p* < 0.01, *** *p* < 0.001, **** *p* < 0.0001, ‘ns’ indicates not significant).

**Figure 5:**
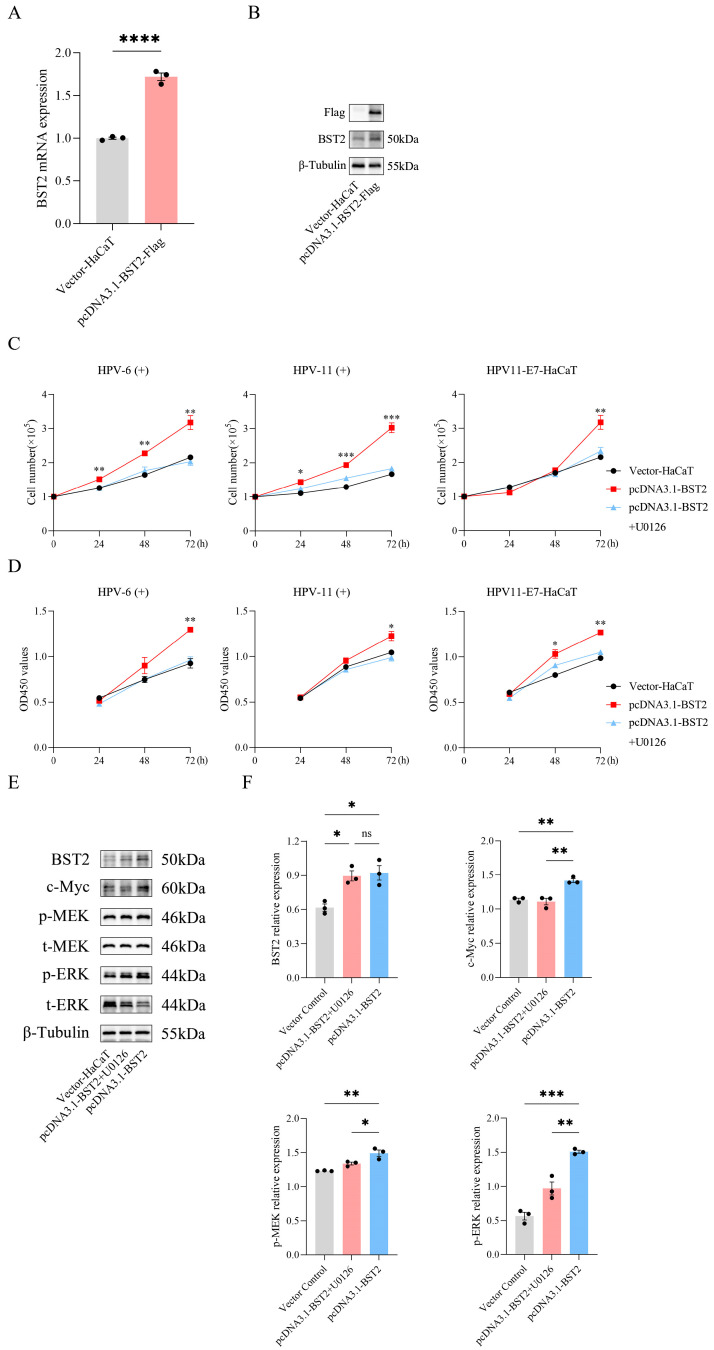
BST2 overexpression promotes keratinocyte proliferation via the MEK/ERK signaling pathway. (**A**) qRT-PCR analysis of BST2 mRNA expression in cells transfected with either vector-HaCaT or pcDNA3.1-BST2-Flag, each dot represents one of 3 independent experiments. (**B**) Western blot analysis of BST2 protein expression following overexpression, with β-Tubulin as a loading control. (**C**) Cell proliferation assays in HPV-6(+), HPV-11(+), and HPV11-E7-HaCaT cells transfected with vector control or BST2 overexpression plasmid, with or without U0126 (MEK inhibitor) treatment. (**D**) CCK-8 assays measuring cell viability at 24, 48, and 72 h post-transfection under the same conditions as (**C**). (**E**) Western blot analysis of BST2, c-Myc, phosphorylated MEK (p-MEK), total MEK (t-MEK), phosphorylated ERK (p-ERK), and total ERK (t-ERK) in cells transfected with vector control or BST2 overexpression plasmid, with or without U0126 treatment. β-Tubulin was used as a loading control. (**F**) Quantification of BST2, c-Myc, p-MEK, and p-ERK protein expression from (**E**), each dot represents one of 3 independent experiments. (* *p* < 0.05, ** *p* < 0.01, *** *p* < 0.001, **** *p* < 0.0001, ‘ns’ indicates not significant).

Previous studies have shown that elevated expression of the interferon-stimulated gene BST2 is strongly linked to excessive tumor cell proliferation in solid tumors, including breast, pancreatic, and gastric cancers [15,16,17]. To further explore whether increased BST2 influences keratinocyte proliferation could affect the proliferation of keratinocytes, we silenced BST2 in HaCaT cells using siRNAs (Figure 4A,B) and performed in vitro proliferation assays. Results showed that BST2 silencing had no notable effect on HaCaT cell apoptosis (Figure S2A–D), but significantly reduced proliferation rates (Figure 4C,D). Conversely, overexpressing BST2 in HaCaT cells via plasmid constructs (Figure 5A,B) markedly enhanced proliferation rates (Figure 5C,D). These findings suggest that low-risk HPV-induced BST2 upregulation significantly promotes keratinocyte proliferation.

### 3.5. Enhanced BST2 Promotes Keratinocyte’s Proliferation by Activating the MEK/ERK/c-Myc Signal Pathway

The above data showed that low-risk HPV-induced BST2 upregulation promotes keratinocyte proliferation, but the underlying mechanism remained unclear. Previous single-gene GSEA analysis suggested that high BST2 expression is associated with positive regulation of the MAPK signaling pathway, known to regulate cell proliferation, apoptosis, and stress responses [19,20,21]. To elucidate how elevated BST2 enhances keratinocyte proliferation, we silenced BST2 in HaCaT cells using siRNAs and observed a significant reduction in the MEK/ERK/c-Myc signaling pathway activity (Figure 4E,F). Conversely, overexpressing BST2 in HaCaT cells via plasmids activated the MEK/ERK/c-Myc pathway, an effect reversed by U0126, a selective MEK1/2 inhibitor (Figure 5E,F). Notably, stimulation with live HPV-6 virus also markedly activated the MEK/ERK/c-Myc pathway (Figure 4E,F). These results demonstrated that the increased BST2 in CA warts promotes keratinocyte proliferation by activating the MEK/ERK/c-Myc signaling pathway.

## 4. Discussion

CA is a prevalent sexually transmitted disease primarily caused by low-risk HPV infection, notably HPV-6 and HPV-11 [1]. Despite its benign nature, CA poses a significant clinical burden due to its high recurrence rate and lack of effective antiviral therapies [5,6]. While high-risk HPV types have been extensively studied, the molecular mechanisms underlying low-risk HPV-induced keratinocyte proliferation remain incompletely understood. Existing research indicates that although low-risk HPV shares many similarities with high-risk HPV in terms of infection process, hijacking host cell protein expression, and mediating host antiviral immune responses, they are not entirely identical [20,21,22,23,24,25]. Differences in viral genome typing ultimately lead to different clinical phenotypes.

In addition to CA, persistent infection with low-risk HPV types, such as HPV-6 and HPV-11, is also associated with an increased risk of carcinoma cervicis uteri (cervical cancer). Cervical cancer remains one of the most prevalent cancers worldwide, and its development is closely linked to chronic HPV infections. Although CA lesions themselves are benign, they share common viral oncogenic mechanisms with cervical cancer, particularly in terms of HPV-induced keratinocyte proliferation and immune modulation. Several studies have demonstrated that HPV vaccination and regular screening can significantly reduce the incidence of cervical cancer by preventing the progression of persistent HPV infections into malignancy [2,9].

Interferon-stimulated genes (ISGs) play a crucial role in the host’s antiviral defense [7,26,27]. As a member of ISGs, BST2 has been reported to be highly associated with excessive cell proliferation and the abnormal activation of signaling pathways such as NF-κB, ERK, and AKT in the development and progression of various tumors, including gastric cancer, liver cancer, and oral squamous cell carcinoma [8,28,29]. However, its role in HPV infection and associated keratinocyte hyperproliferation has remained unexplored. In this study, we identified BST2 as a significantly upregulated gene in CA lesions and demonstrated its role in promoting keratinocyte proliferation through the MEK/ERK/c-Myc signaling pathway.

Further analysis of BST2 protein expression revealed that its monomer (~40 kDa) exhibited low abundance with no significant differences between groups, whereas the dimeric form (~50 kDa) was more prevalent and showed marked differences. The literature suggests that BST2 undergoes extensive glycosylation at conserved asparagine residues (-Asn65, 92) and forms disulfide bonds at cysteine residues (-Cys53, 63, 91), stabilizing its dimeric structure. Given the inherent instability of BST2 monomers, dimerization is essential for maintaining structural integrity and functional activity [30,31,32]. X-ray crystallography studies have further demonstrated that BST2 monomers in both humans and mice exhibit dynamic instability due to the coiled-coil structure of the extracellular domain. The formation of inter-molecular disulfide bonds between monomers is necessary to maintain structural stability, thereby enabling its functional activity [33,34,35]. Therefore, our study focused exclusively on the BST2 dimer for subsequent experiments.

Mechanistically, our findings suggest that BST2 upregulation in HPV-infected keratinocytes is mediated through the cGAS/STING pathway. Knockdown of BST2 significantly reduced keratinocyte proliferation, while its overexpression enhanced proliferation via activation of the MEK/ERK/c-Myc pathway. This highlights BST2 not only as a key effector in HPV-induced keratinocyte hyperplasia but also as a potential link between innate immune signaling and abnormal cell proliferation in CA.

Although BST2 is known for its antiviral functions, whether it exerts similar effects on HPV remains unclear. Unlike enveloped viruses such as HIV, HPV is a DNA virus that lacks a lipid membrane, making BST2’s classical antiviral mechanism less applicable [36]. Instead, we speculate that BST2 may influence HPV pathogenesis through immunomodulatory interactions within the local microenvironment or by regulating other ISGs. Future studies should explore whether BST2 contributes to immune evasion in CA lesions and whether targeting BST2 could modulate the host response to HPV infection.

The findings of this study highlight BST2 as a potential novel therapeutic target for condyloma acuminata (CA). Given that BST2 plays a critical role in promoting keratinocyte proliferation in HPV-infected tissues, targeting BST2 could be an effective strategy to inhibit keratinocyte proliferation, potentially reducing recurrence rates of CA lesions. This is particularly relevant as existing treatments primarily focus on symptom management, such as wart removal, but often fail to address the underlying molecular mechanisms of HPV-induced proliferation.

Moreover, BST2-based therapies could be developed, such as small molecule inhibitors or monoclonal antibodies, which could complement existing treatments, especially in cases of recurrent or treatment-resistant CA. These therapies could target the BST2-mediated signaling pathway to control abnormal cell proliferation and promote therapeutic efficacy in patients with persistent or recurrent lesions.

Additionally, our study provides important insights into the molecular mechanisms that underlie abnormal keratinocyte proliferation in HPV-infected tissues, which could have broader implications for HPV-related disease management. The insights gained from this study could inform the development of new therapeutic approaches for other HPV-associated lesions, including oral, anal, and cervical warts, expanding the potential clinical applications of BST2-targeted therapies.

In light of the findings from this study, it is crucial to recognize the importance of sex education and public health initiatives in the prevention of HPV-related diseases. Education on safe sexual practices, the benefits of HPV vaccination, and regular screening programs are essential in reducing the transmission of HPV and preventing the development of not only condyloma acuminata but also more severe consequences, such as cervical cancer. Evidence suggests that sexual education programs that incorporate information on HPV vaccination and the importance of early detection through HPV testing can significantly reduce the burden of cervical cancer globally [4,37].

In conclusion, our study identifies BST2 as a critical regulator of keratinocyte proliferation in CA, acting through the MEK/ERK/c-Myc signaling pathway. These findings provide novel insights into the molecular mechanisms underlying low-risk HPV-induced keratinocyte hyperproliferation and suggest BST2 as a potential therapeutic target for CA. Further research is warranted to investigate its broader role in HPV-associated skin diseases and its potential for therapeutic intervention.

## 5. Conclusions

This study reveals that low-risk HPV induces BST2 via the cGAS/STING pathway, which drives keratinocyte proliferation through MEK/ERK/c-Myc signaling. BST2 may represent a novel therapeutic target for condyloma acuminata.

## Data Availability

The data presented in this study are openly available in GEO database.

## Supplementary Materials

The following supporting information can be downloaded at https://www.mdpi.com/article/10.3390/biomedicines14020339/s1. Figure S1: Lentiviral infection of Vector-HaCaT and HPV11-E7-HaCaT cells observed under a fluorescence microscope. (A) Bright-field (BF) and GFP fluorescence images of HPV11-E7-HaCaT cells at 100× (top) and 200× (bottom) magnification. (B) Bright-field (BF) and GFP fluorescence images of Vector-HaCaT cells at 100× (top) and 200× (bottom) magnification; Figure S2: Silencing BST2 does not significantly affect apoptosis in the condyloma acuminatum cell model. (A) Apoptosis analysis of HaCaT cells infected with HPV-6 live virus 24 h after BST2 silencing, assessed using Annexin V-APC/PI double staining. (B) Apoptosis analysis of HaCaT cells infected with HPV-11 live virus 24 h after BST2 silencing, assessed using Annexin V-APC/PI double staining. (C) Apoptosis analysis of the blank control group using Annexin V-APC/PI double staining. (D) Statistical analysis of apoptosis rates among the siNC, siBST2#1, and siBST2#2 groups. “ns” indicates *p* ≥ 0.05, not statistically significant; Table S1: List of siRNA products and target sequences used in this study. This table provides the product names and corresponding oligonucleotide sequences for siRNAs targeting BST2 and STING, which were applied for gene-silencing experiments; Table S2: Primer sequences used for quantitative real-time PCR (qPCR). This table summarizes the forward and reverse primer sequences for the indicated target genes, including ACTB and BST2, used to quantify gene expression levels.

## Abbreviations

The following abbreviations are used in this manuscript:

APC	Allophycocyanin
BST2	Bone marrow stromal cell antigen 2
CA	Condyloma acuminata
CCK-8	Cell Counting Kit-8
cGAS	Cyclic GMP–AMP synthase
DEGs	Differentially expressed genes
GEO	Gene Expression Omnibus
HPV	Human papillomavirus
ISGs	Interferon-stimulated genes
PAMP	Pathogen-associated molecular pattern
PRRs	Pattern recognition receptors
STING	Stimulator of interferon genes

## References

[1] Grennan D. (2019). Genital warts. JAMA.

[2] Bhattacharjee R., Das S.S., Biswal S.S., Nath A., Das D., Basu A., Malik S., Kumar L., Kar S., Singh S.K. (2022). Mechanistic role of HPV-associated early proteins in cervical cancer: Molecular pathways and targeted therapeutic strategies. Crit. Rev. Oncol./Hematol..

[3] Sindhuja T., Bhari N., Gupta S. (2022). Asian guidelines for condyloma acuminatum. J. Infect. Chemother..

[4] Plotzker R.E., Vaidya A., Pokharel U., Stier E.A. (2023). Sexually transmitted human papillomavirus: Update in epidemiology, prevention, and management. Infect. Dis. Clin. North Am..

[5] Widschwendter A., Böttcher B., Riedl D., Coban S., Mutz-Dehbalaie I., Matteucci Gothe R., Ciresa-König A., Marth C., Fessler S. (2019). Recurrence of genitals warts in pre-HPV vaccine era after laser treatment. Arch. Gynecol. Obstet..

[6] Giuliano A.R., Sirak B., Abrahamsen M., Silva R.J.C., Baggio M.L., Galan L., Cintra R.C., Lazcano-Ponce E., Villa L.L. (2019). Genital wart recurrence among men residing in Brazil, Mexico, and the United States. J. Infect. Dis..

[7] Mesev E.V., LeDesma R.A., Ploss A. (2019). Decoding type I and III interferon signalling during viral infection. Nat. Microbiol..

[8] Hopfner K.P., Hornung V. (2020). Molecular mechanisms and cellular functions of cGAS–STING signalling. Nat. Rev. Mol. Cell Biol..

[9] Doorbar J., Quint W., Banks L., Bravo I.G., Stoler M., Broker T.R., Stanley M.A. (2012). The biology and life-cycle of human papillomaviruses. Vaccine.

[10] Xie J., Zhang P., Crite M., DiMaio D. (2020). Papillomaviruses go retro. Pathogens.

[11] Ivashkiv L.B., Donlin L.T. (2014). Regulation of type I interferon responses. Nat. Rev. Immunol..

[12] Au-Yeung N., Horvath C.M. (2018). Transcriptional and chromatin regulation in interferon and innate antiviral gene expression. Cytokine Growth Factor Rev..

[13] Timofeev O., Giron P., Lawo S., Pichler M., Noeparast M. (2024). ERK pathway agonism for cancer therapy: Evidence, insights, and a target discovery framework. npj Precis. Oncol..

[14] Song Y., Bi Z., Liu Y., Qin F., Wei Y., Wei X. (2023). Targeting RAS–RAF–MEK–ERK signaling pathway in human cancer: Current status in clinical trials. Genes Dis..

[15] Harada T., Ozaki S., Oda A., Tsuji D., Ikegame A., Iwasa M., Udaka K., Fujii S., Nakamura S., Miki H. (2013). Combination with a defucosylated anti-HM1.24 monoclonal antibody plus lenalidomide induces marked ADCC against myeloma cells and their progenitors. PLoS ONE.

[16] Ozaki S., Kosaka M., Wakatsuki S., Abe M., Koishihara Y., Matsumoto T. (1997). Immunotherapy of multiple myeloma with a monoclonal antibody directed against a plasma cell-specific antigen, HM1.24. Blood.

[17] Mahauad-Fernandez W.D., Okeoma C.M. (2017). Cysteine-linked dimerization of BST-2 confers anoikis resistance to breast cancer cells by negating proapoptotic activities to promote tumor cell survival and growth. Cell Death Dis..

[18] Zheng C., Wang J., Zhou Y., Duan Y., Zheng R., Xie Y., Wei X., Wu J., Shen H., Ye M. (2024). IFNα-induced BST2+ tumor-associated macrophages facilitate immunosuppression and tumor growth in pancreatic cancer by ERK–CXCL7 signaling. Cell Rep..

[19] Mukai S., Oue N., Oshima T., Mukai R., Tatsumoto Y., Sakamoto N., Sentani K., Tanabe K., Egi H., Hinoi T. (2017). Overexpression of transmembrane protein BST2 is associated with poor survival of patients with esophageal, gastric, or colorectal cancer. Ann. Surg. Oncol..

[20] Sudarshan S.R., Schlegel R., Liu X. (2022). Two conserved amino acids differentiate the biology of high-risk and low-risk HPV E5 proteins. J. Med. Virol..

[21] Nasiri-Aghdam M., Garcia-Chagollan M., Pereira-Suarez A.L., Aguilar-Lemarroy A., Jave-Suarez L.F. (2023). Splicing characterization and isoform switch events in human keratinocytes carrying oncogenes from high-risk HPV-16 and low-risk HPV-84. Int. J. Mol. Sci..

[22] Jones R.N., Miyauchi S., Roy S., Boutros N., Mayadev J.S., Mell L.K., Califano J.A., Venuti A., Sharabi A.B. (2024). Computational and AI-driven 3D structural analysis of human papillomavirus (HPV) oncoproteins E5, E6, and E7 reveal significant divergence of HPV E5 between low-risk and high-risk genotypes. Virology.

[23] Aranda-Rivera A.K., Cruz-Gregorio A., Briones-Herrera A., Pedraza-Chaverri J. (2021). Regulation of autophagy by high- and low-risk human papillomaviruses. Rev. Med. Virol..

[24] Murakami I., Egawa N., Griffin H., Yin W., Kranjec C., Nakahara T., Kiyono T., Doorbar J. (2019). Roles for E1-independent replication and E6-mediated p53 degradation during low-risk and high-risk human papillomavirus genome maintenance. PLoS Pathog..

[25] Thomas M., Banks L. (2021). The biology of papillomavirus PDZ associations: What do they offer papillomaviruses?. Curr. Opin. Virol..

[26] Streicher F., Jouvenet N. (2019). Stimulation of innate immunity by host and viral RNAs. Trends Immunol..

[27] Wirusanti N.I., Baldridge M.T., Harris V.C. (2022). Microbiota regulation of viral infections through interferon signaling. Trends Microbiol..

[28] Shan F., Shen S., Wang X., Chen G. (2023). BST2 regulated by the transcription factor STAT1 can promotes metastasis, invasion and proliferation of oral squamous cell carcinoma via the AKT/ERK1/2 signaling pathway. Int. J. Oncol..

[29] Liu W., Cao Y., Guan Y., Zheng C. (2018). BST2 promotes cell proliferation, migration and induces NF-κB activation in gastric cancer. Biotechnol. Lett..

[30] Welbourn S., Kao S., Du Pont K.E., Andrew A.J., Berndsen C.E., Strebel K. (2015). Positioning of cysteine residues within the N-terminal portion of the BST-2/tetherin ectodomain is important for functional dimerization of BST-2. J. Biol. Chem..

[31] Arias J.F., Iwabu Y., Tokunaga K. (2011). Structural basis for the antiviral activity of BST-2/tetherin and its viral antagonism. Front. Microbiol..

[32] Andrew A.J., Miyagi E., Kao S., Strebel K. (2009). The formation of cysteine-linked dimers of BST-2/tetherin is important for inhibition of HIV-1 virus release but not for sensitivity to Vpu. Retrovirology.

[33] Schubert H.L., Zhai Q., Sandrin V., Eckert D.M., Garcia-Maya M., Saul L., Sundquist W.I., Steiner R.A., Hill C.P. (2010). Structural and functional studies on the extracellular domain of BST2/tetherin in reduced and oxidized conformations. Proc. Natl. Acad. Sci. USA.

[34] Hinz A., Miguet N., Natrajan G., Usami Y., Yamanaka H., Renesto P., Hartlieb B., McCarthy A.A., Simorre J.P., Göttlinger H. (2010). Structural basis of HIV-1 tethering to membranes by the BST-2/tetherin ectodomain. Cell Host Microbe.

[35] Yang H., Wang J., Jia X., McNatt M.W., Zang T., Pan B., Meng W., Wang H.W., Bieniasz P.D., Xiong Y. (2010). Structural insight into the mechanisms of enveloped virus tethering by tetherin. Proc. Natl. Acad. Sci. USA.

[36] Neil S.J., Zang T., Bieniasz P.D. (2008). Tetherin inhibits retrovirus release and is antagonized by HIV-1 Vpu. Nature.

[37] Reuschenbach M., Doorbar J., Del Pino M., Joura E.A., Walker C., Drury R., Rauscher A., Saah A.J. (2023). Prophylactic HPV vaccines in patients with HPV-associated diseases and cancer. Vaccine.

