# Supplementary material for: cGAS-STING Pathway-Induced BST2 Enhances HPV-Infected Keratinocyte Proliferation in Condyloma Acuminata
Source: Biomedicines. 2026 Feb 1;14(2):339. doi: 10.3390/biomedicines14020339 (PMC12938457; doi:10.3390/biomedicines14020339)
Supplement: Supplementary file 1 [file biomedicines-14-00339-s001.zip › Table S1+S2.pdf]

Sup. Tab. S1

Target gene	Product name	Sequence
	genOFFTM st-h-BST2_001	GAATCGCGGACAAGAAGTA
BST2	genOFFTM st-h-BST2_002	CCTTGATTATCTTCACCAT
	genOFFTM st-h-BST2_003	GAGAGATCACTACATTAAA
	genOFFTM st-h-STING_001	GGAAGGAGTTTGAAGGAAATT
STING	genOFFTM st-h-STING_002	GGACATCTTTGAGAATGAATT
	genOFFTM st-h-STING_003	GCAGCAGATTGTCTTATATT

Sup. Tab. S2

Target Gene	Forward Sequence	Reverse Sequence
ACTB	CACCATTGGCAATGAGCGGTTC	AGGTCTTTGCGGATGTCCACGT
BST2	TCTCCTGCAACAAGAGCTGACC	TCTCTGCATCCAGGGAAGCCAT
