# Supplementary figures and images for: cGAS-STING Pathway-Induced BST2 Enhances HPV-Infected Keratinocyte Proliferation in Condyloma Acuminata
Source: Biomedicines. 2026 Feb 1;14(2):339. doi: 10.3390/biomedicines14020339 (PMC12938457; doi:10.3390/biomedicines14020339)

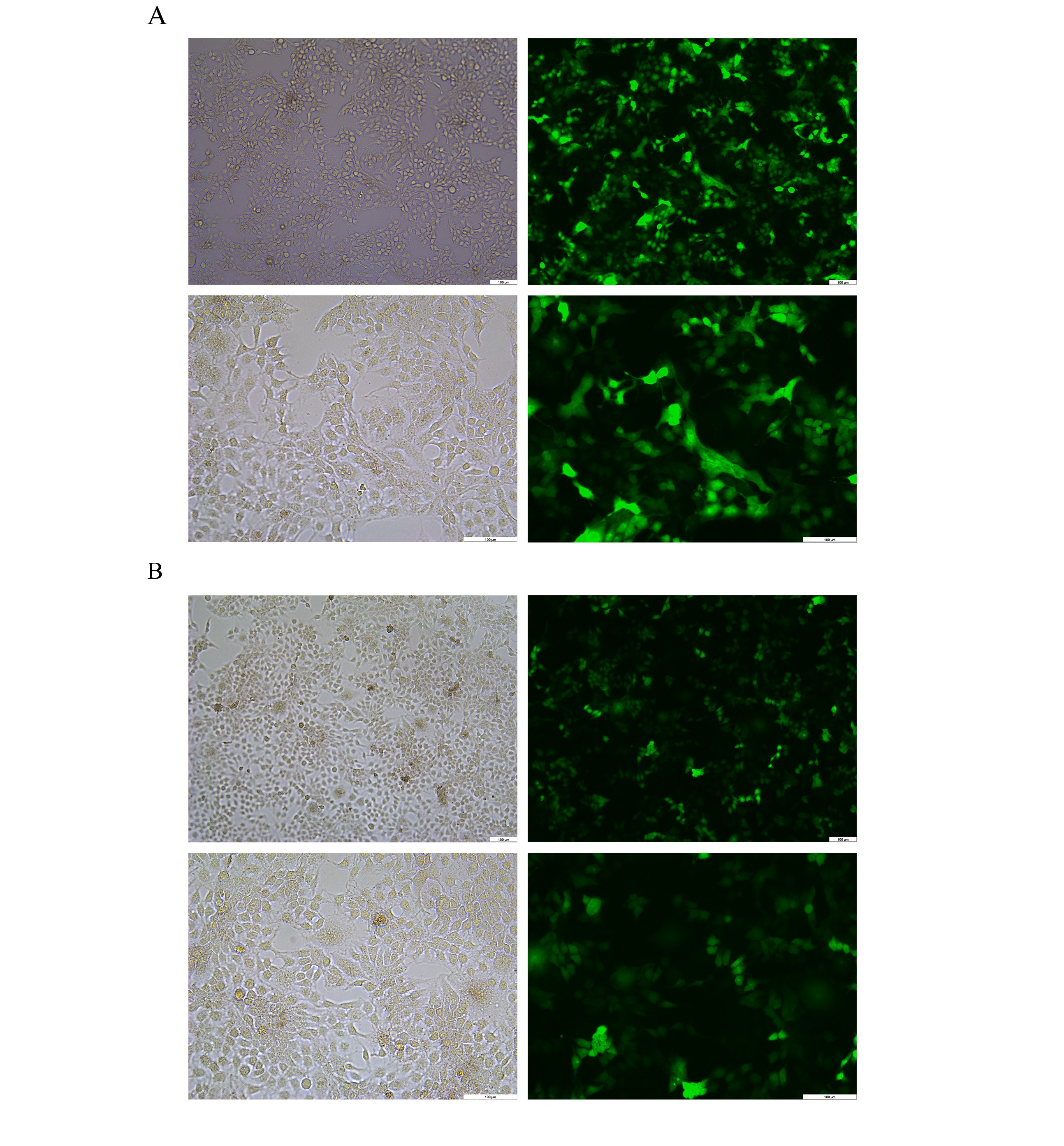

Supplement: Supplementary file 1 [file biomedicines-14-00339-s001.zip › Figure S1.jpg]

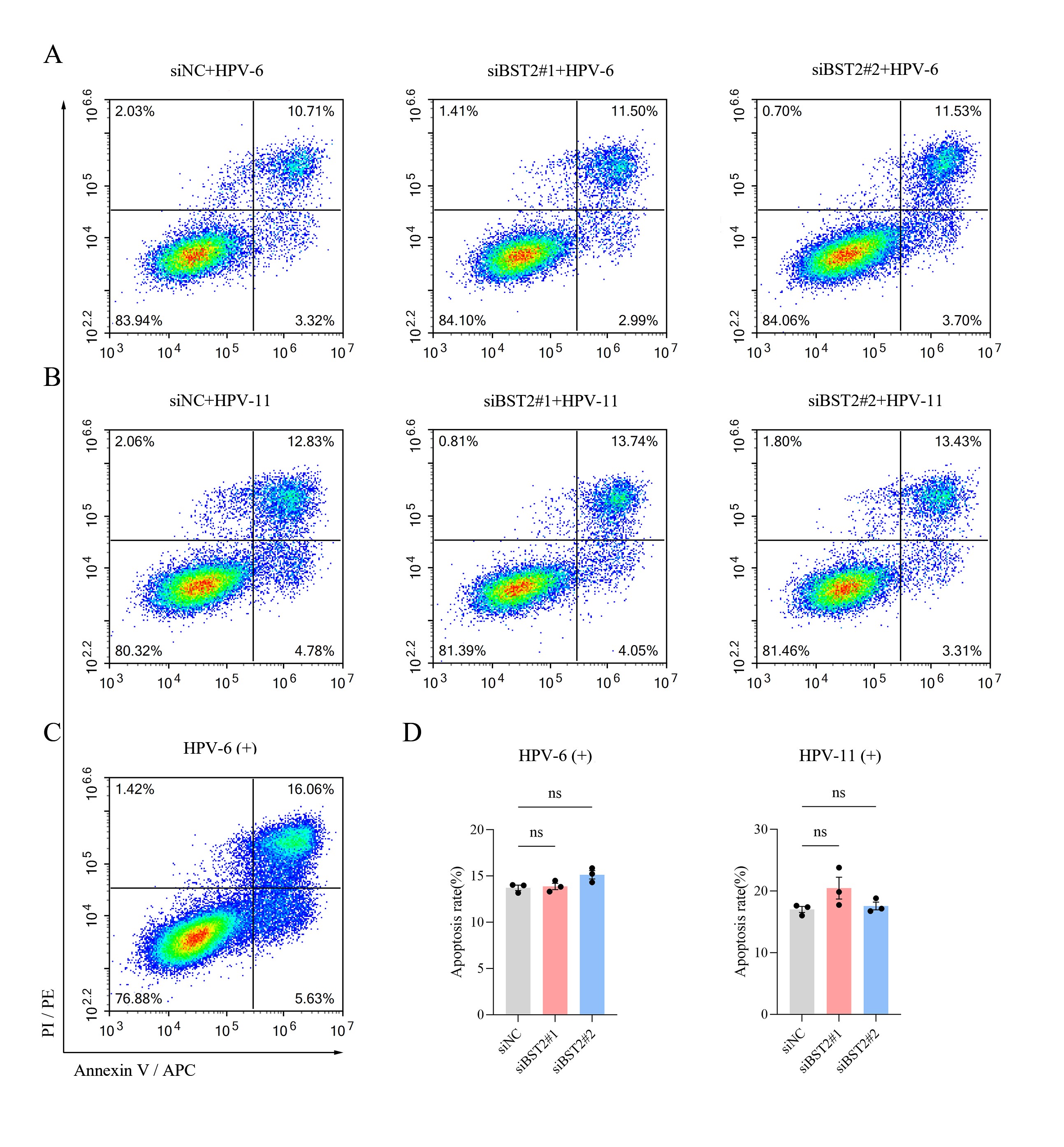

Supplement: Supplementary file 1 [file biomedicines-14-00339-s001.zip › Figure S2.jpg]
